# Association of composite liver function indices with trauma-induced coagulopathy in adult trauma patients

**DOI:** 10.3389/fsurg.2026.1860161

**Published:** 2026-10-23

**Authors:** Jiejun Hu, Xinxin Chen, Jiao Bao, Weichao Lu, Manzhen Ying

**Affiliations:** Dongyang Hospital of Wenzhou Medical University, Dongyang/Jinhua, Zhejiang, China

**Keywords:** adult trauma, ALBI score, composite liver function indices, critical trauma, trauma-induced coagulopathy

## Abstract

**Objective:**

Trauma-induced coagulopathy (TIC) is a life-threatening complication associated with uncontrolled hemorrhage and high mortality in critically ill trauma patients. This study aimed to evaluate the associations and discriminative performance of four composite liver-function indices—ALBI, TBil/ALB, DBil/TBil, and GGT/ALP—with TIC-related outcomes in adult trauma patients.

**Methodology:**

This single-center retrospective cohort study included 634 adult trauma patients. Laboratory data were collected at baseline, 24 h after admission, and 48 h after admission. The primary outcome was the continuous ISTH DIC score assessed 48 h after admission. Overt DIC, non-overt DIC, and ATC were analyzed as secondary or exploratory TIC-related outcomes. Multivariable models adjusted for age, sex, admission lactate, admission pH, admission temperature, traumatic brain injury, hepatic injury, and surgery. Associations between each index and TIC-related outcomes were examined using multivariable linear and logistic regression analyses.

**Results:**

ALBI showed the most consistent associations with TIC-related outcomes and the strongest index-alone discrimination for non-overt DIC among the evaluated indices. The TBil/ALB ratio retained independent predictive value, though with somewhat lower overall consistency. The DBil/TBil and GGT/ALP ratios, by contrast, exhibited weaker associations that varied across time points and outcome definitions. Serial measurements over the first 24 h contributed incremental prognostic information; however, no dynamic index outperformed the baseline ALBI score in predictive strength.

**Conclusion:**

ALBI was consistently associated with TIC-related outcomes assessed 48 h after admission, and this association remained stable across the examined clinical subgroups. ALBI may serve as a readily available adjunctive risk marker for early coagulopathy risk stratification. Prospective validation is warranted before clinical implementation.

## 1 Introduction

Trauma remains a leading cause of death worldwide, and uncontrolled hemorrhage is still one of the most important preventable causes of early mortality after severe injury (1). A key contributor to this process is trauma-induced coagulopathy (TIC), a complex and rapidly evolving disorder involving impaired clot formation, dysregulated fibrinolysis, platelet dysfunction, endothelial injury, and shock-related hypoperfusion (1, 2). TIC may be present on admission or develop early during resuscitation, and is consistently associated with greater transfusion requirements, organ failure, and death (1, 2). Recent evidence from a Pakistani trauma cohort confirmed that abnormal coagulation profiles, including prolonged PT and APTT, were significantly correlated with higher injury severity scores, underscoring the global relevance of early coagulation assessment in trauma care (3). Therefore, early identification of patients at high risk for TIC is clinically important for timely hemostatic resuscitation, intensified monitoring, and damage-control decision-making.

Current approaches to TIC recognition remain imperfect. Conventional coagulation tests, such as prothrombin time, activated partial thromboplastin time, fibrinogen, and platelet count, are widely available and clinically useful, but they reflect only selected components of the coagulation system and may not fully capture the dynamic and heterogeneous nature of TIC (1, 4). Viscoelastic assays may provide a more comprehensive assessment, yet they are not universally available, particularly in resource-constrained or time-sensitive settings (4). Accordingly, there is a continuing need for simple, objective, and readily obtainable biomarkers that can support early TIC risk stratification using routine laboratory data.

The liver is central to hemostatic homeostasis because it synthesizes most procoagulant and anticoagulant factors, regulates fibrinolytic proteins, and contributes to the clearance of activated hemostatic mediators (5, 6). In acute trauma, albumin and bilirubin may reflect not only hepatic synthetic or excretory function, but also systemic inflammation, hemodilution, capillary leak, hypoperfusion, hemolysis, and transfusion-related changes. Therefore, composite liver-function indices may be better viewed as liver-function-related and systemic stress markers rather than direct measures of hepatic reserve. Among these indices, the albumin-bilirubin (ALBI) score is an objective and reproducible measure derived from routine laboratory parameters and has evolved from a tool used in hepatocellular carcinoma to a more general indicator of liver function (7). Other ratio-based indices, including the total bilirubin-to-albumin (TBil/ALB) ratio, direct-to-total bilirubin (DBil/TBil) ratio, and gamma-glutamyl transferase-to-alkaline phosphatase (GGT/ALP) ratio, may also reflect distinct patterns of hepatic dysfunction, but their relevance in trauma-associated coagulopathy remains unclear.

To date, evidence directly comparing these composite liver function indices for TIC prediction is limited. In particular, it remains uncertain which marker is the most robust across different time windows and clinically relevant TIC phenotypes. We compared these indices at admission, 24 h after admission, and as 0–24-hour changes in relation to TIC-related outcomes assessed 48 h after admission. Admission values were considered the primary exposure window, whereas 24-hour and delta values were interpreted as exploratory dynamic indicators.

## 2 Methods

### 2.1 Study design and population

This retrospective cohort study was conducted at a Grade A tertiary hospital in Zhejiang Province. The study protocol was approved by the Institutional Review Board, with ethics approval number ″东人医2026-YX−066″. We screened adult trauma patients treated at the hospital. Patients were eligible for inclusion if they met all of the following criteria: (1) age ≥ 18 years; (2) a primary diagnosis of trauma; (3) availability of hospital-based trauma care records; and (4) availability of liver-function and coagulation parameters required for the construction of exposure variables and outcome assessments. Patients with pre-existing liver cirrhosis or chronic hepatic failure, missing baseline liver-function data, or missing coagulation data required for ISTH DIC scoring 48 h after admission were excluded. Patients with severe baseline coagulopathy (PT >20 s or PLT <50 × 10⁹/L at baseline) were included in the primary analysis; their exclusion was examined in a sensitivity analysis.

### 2.2 Data collection and variable definitions

Clinical and demographic variables included age, sex, admission lactate, admission pH, admission temperature, traumatic brain injury, hepatic injury, and surgical intervention records. These variables were selected as clinically relevant covariates available in the expanded electronic database and were used for adjusted analyses.

Laboratory parameters were collected at three predefined time points: admission (0 h, T1), 24 h (T2), and 48 h (T3). Liver function tests included alanine aminotransferase (ALT), aspartate aminotransferase (AST), total bilirubin (TBil), direct bilirubin (DBil), indirect bilirubin (IBil), albumin (ALB), globulin (GLB), gamma-glutamyl transferase (GGT), and alkaline phosphatase (ALP). Coagulation profiles included PT, activated partial thromboplastin time (APTT), thrombin time (TT), fibrinogen (FIB), D-dimer, and PLT.

Four composite liver function indices were calculated as the primary exposure variables, as detailed below:

ALBI Score: [log10 TBil (µmol/L) × 0.66] + [ALB (g/L) × −0.085] (8).
TBil/ALB Ratio: TBil/ALB.
GGT/ALP Ratio: GGT/ALP.
DBil/TBil Ratio: DBil/TBil.

ALBI score, TBil/ALB ratio, DBil/TBil ratio, and GGT/ALP ratio were calculated from the corresponding raw liver-function parameters. In the acute trauma setting, these indices were considered liver-function-related composite markers that may also reflect systemic stress, inflammation, hemodilution, capillary leak, hypoperfusion, and transfusion-related changes.

Admission values were considered the primary exposure window. The 24 h values and 0–24 h changes were analyzed as exploratory dynamic indicators reflecting early in-hospital evolution rather than pre-treatment risk stratification.

### 2.3 Outcome measures

The primary outcome was the continuous ISTH DIC score at 48 h. International Society on Thrombosis and Haemostasis scoring system (PLT, D-dimer, prolonged PT, FIB; range 0–8) (9). Secondary binary outcomes included overt DIC, defined as ISTH DIC score ≥5, and acute traumatic coagulopathy (ATC), defined as PTr >1.2 (10). Non-overt DIC, defined as ISTH DIC score ≥4 (11), was analyzed as an exploratory early coagulopathy phenotype.

PTr was calculated as PT divided by the local laboratory reference PT value of 12.5 s.

### 2.4 Statistical analysis

Continuous variables were presented as mean ± standard deviation (SD) or median with interquartile range (IQR), depending on their distribution as assessed by the Shapiro–Wilk test. Categorical variables were expressed as frequencies and percentages. The temporal trajectories of laboratory parameters across the three time points (0 h, 24 h, 48 h) were analyzed using the Friedman test for repeated measures.

Associations between composite liver-function indices and 48-hour TIC-related outcomes were assessed using linear regression for the continuous ISTH DIC score and logistic regression for binary outcomes. Three models were fitted for each index: Model 1 was unadjusted; Model 2 was adjusted for age and sex; and Model 3 was adjusted for age, sex, admission lactate, admission pH, admission temperature, traumatic brain injury, hepatic injury, and surgical intervention records. Each composite index was entered separately to avoid multicollinearity arising from shared components.

ROC curves were generated using each composite index alone to evaluate index-alone discrimination, and incremental discrimination analyses compared admission ALBI alone, the clinical model alone, and the clinical model plus admission ALBI. The Area Under the Curve (AUC) with 95% confidence intervals (CI) was calculated. When applicable, cutoff analyses were considered exploratory and were not used to define clinical decision thresholds. Optimal cutoffs were estimated using the Youden index and were interpreted as exploratory thresholds rather than clinical decision cutoffs. For the continuous outcome (ISTH DIC score), model performance was evaluated using the coefficient of determination (R-squared), adjusted R-squared, root mean square error (RMSE), and the Akaike Information Criterion (AIC).

Given the limited sample size, subgroup analyses were performed by introducing multiplicative interaction terms (exposure ×  subgroup variable) into the fully adjusted models, rather than stratifying the cohort. Exploratory subgroup analyses were performed for admission ALBI and non-overt DIC using interaction terms between ALBI and prespecified subgroup variables, including age, sex, lactate, traumatic brain injury, hepatic injury, surgical intervention record, hypothermia, and acidosis. These analyses were considered exploratory and were not interpreted as confirmed effect modification. The *P*-value for the interaction term was used to assess effect modification. To account for the eight interaction tests, nominal *P* values were adjusted using the Bonferroni method. Benjamini–Hochberg false-discovery-rate-adjusted *P* values were also calculated.

Sensitivity analyses were conducted for ALBI across TIC-related outcomes using the main complete-case analysis, exclusion of patients with severe baseline coagulopathy, and single-imputation analysis for selected covariates with missing values. Single imputation used median imputation for continuous variables and mode imputation for sex. These analyses were interpreted as supportive rather than confirmatory. Given the exploratory nature of multiple comparisons, emphasis was placed on effect size, confidence intervals, and consistency of associations rather than isolated *P* values.

All statistical analyses were performed using Python (version 3.11) with the statsmodels, scikit-learn, and scipy packages. A two-sided *P*-value < 0.05 was considered statistically significant.

## 3 Results

### 3.1 Baseline characteristics and temporal trajectories

After excluding patients younger than 18 years, the final analytic cohort included 634 adult trauma patients. Among them, 172 developed non-overt DIC, 63 developed overt DIC, and 180 met the definition of ATC. Baseline was defined as the time of admission, 24-hour measurements were obtained 24 h after admission, and 48-hour measurements were obtained 48 h after admission.

Temporal patterns of liver-function and coagulation parameters are presented in Table 1. From admission to 48 h after admission, several liver enzymes and composite liver-function indices changed significantly, including ALT, AST, GGT, ALP, ALBI, TBil/ALB, DBil/TBil, and GGT/ALP. The median ALBI score was −2.06 at admission, −2.02 at 24 h after admission, and −2.06 at 48 h after admission (*P* < 0.001). TBil remained largely stable across the three time points, whereas ALB showed a statistically significant but clinically modest temporal change. In contrast, coagulation parameters displayed more pronounced variation over time. Among the 142 patients with serial platelet measurements, PLT declined from 194.00 × 10⁹/L at admission to 130.00 × 10⁹/L at 24 h and 108.50 × 10⁹/L at 48 h (*P* < 0.001). D-dimer decreased over time, while FIB increased substantially from 2.57 g/L at admission to 3.48 g/L at 24 h and 5.15 g/L at 48 h after admission (*P* < 0.001). PT, APTT, and TT also changed significantly across the three time points. Platelet counts at T1 and T2 were not measured in 492 of 634 patients; T3 platelet counts were complete, and the main analyses did not use T1/T2 platelet data. Missingness patterns for key variables are shown in Supplementary Table S7. The comparatively modest changes in ALBI may reflect its integration of broader hepatic and systemic processes, whereas conventional coagulation measures can change rapidly in response to factor consumption, resuscitation, and the acute-phase response.

**Table 1:** Temporal trajectories of liver-function and coagulation parameters in the adult cohort.

Variable	Admission	24 h	48 h	*P* for overall change
ALT	23.00 (16.00, 38.75)	24.00 (16.25, 40.75)	31.00 (18.00, 53.00)	0.001*
AST	38.00 (27.00, 61.00)	34.00 (23.00, 57.00)	34.00 (22.00, 54.00)	<0.001*
GGT	19.00 (14.00, 30.00)	23.00 (15.00, 45.00)	36.00 (21.00, 74.00)	<0.001*
ALP	55.50 (44.00, 69.00)	62.00 (50.00, 75.00)	71.00 (56.00, 92.00)	<0.001*
TBIL	15.20 (11.40, 20.80)	15.20 (11.60, 20.50)	15.30 (11.00, 21.20)	0.662
DBIL	5.60 (4.20, 7.80)	5.60 (4.10, 7.80)	5.50 (3.90, 8.40)	0.781
IBIL	9.40 (7.10, 13.38)	9.60 (7.10, 12.78)	8.90 (6.80, 12.43)	0.022*
ALB	33.40 (29.90, 36.70)	33.00 (30.00, 36.00)	33.50 (30.60, 36.90)	<0.001*
GLB	20.70 (17.62, 23.80)	23.40 (20.80, 26.20)	25.60 (22.50, 28.60)	<0.001*
ALBI	−2.06 (−2.33, −1.77)	−2.02 (−2.26, −1.77)	−2.06 (−2.39, −1.81)	<0.001*
TBil/ALB	0.47 (0.36, 0.62)	0.46 (0.35, 0.63)	0.44 (0.32, 0.64)	0.015*
DBil/TBil	0.37 (0.32, 0.42)	0.37 (0.32, 0.42)	0.38 (0.33, 0.43)	<0.001*
GGT/ALP	0.36 (0.24, 0.58)	0.40 (0.26, 0.69)	0.53 (0.32, 0.96)	<0.001*
PLT	194.00 (160.25, 242.75)	130.00 (86.25, 171.75)	125.00 (91.00, 165.75)	<0.001*
D_dimer	11.91 (5.42, 20.00)	5.23 (2.51, 12.80)	3.90 (2.14, 7.87)	<0.001*
PT	14.20 (13.40, 15.30)	14.90 (13.90, 16.00)	14.20 (13.40, 15.20)	<0.001*
FIB	2.57 (1.92, 3.36)	3.48 (2.44, 4.67)	5.15 (3.94, 6.20)	<0.001*
APTT	35.50 (32.10, 39.48)	38.20 (35.00, 41.80)	39.30 (35.92, 43.30)	<0.001*
TT	16.00 (15.00, 17.10)	15.10 (14.10, 16.30)	14.70 (14.00, 15.50)	<0.001*

Values are median (interquartile range) based on all available observations at each time point. *P* values were calculated using the Friedman test among patients with measurements at all three time points for each variable. For PLT, descriptive statistics use the available observations at each time point (admission and 24 h: *n* = 142; 48 h: *n* = 634), whereas the *P* value is based on the 142 patients with serial measurements, whose median PLT was 194.00 × 10⁹/L at admission, 130.00 × 10⁹/L at 24 h, and 108.50 × 10⁹/L at 48 h. Missingness for all variables is detailed in Supplementary Table S7. *P < 0.05.

### 3.2 Independent associations between liver function indices and coagulopathy

The fully adjusted associations between composite liver-function indices and TIC-related outcomes are shown in Table 2. Model 3 adjusted for age, sex, lactate at admission, pH at admission, temperature at admission, traumatic brain injury, hepatic injury, and surgery. Detailed results for Models 1 through 3 are provided in Supplementary Table S1, and model-fitting metrics are summarized in Supplementary Table S2.

**Table 2:** Associations of liver-function composite indices with TIC-related outcomes in the fully adjusted model.

Outcome	Index	Time	N	Events	Effect	Effect (95% CI)	*P*
ISTH DIC score	ALBI	Admission	526		β per 1-SD	0.470 (0.378*–*0.561)	<0.001*
	ALBI	24 h	517		β per 1-SD	0.384 (0.292–0.477)	<0.001*
	ALBI	Delta	517		β per 1-SD	−0.116 (−0.214– −0.019)	0.02*
	TBil/ALB	Admission	526		β per 1-SD	0.312 (0.221–0.403)	<0.001*
	TBil/ALB	24 h	517		β per 1-SD	0.418 (0.329–0.507)	<0.001*
	TBil/ALB	Delta	517		β per 1-SD	0.112 (0.015–0.210)	0.023*
	DBil/TBil	Admission	526		β per 1-SD	0.295 (0.202–0.389)	<0.001*
	DBil/TBil	24 h	517		β per 1-SD	0.280 (0.186–0.375)	<0.001*
	DBil/TBil	Delta	517		β per 1-SD	−0.018 (−0.114–0.079)	0.719
	GGT/ALP	Admission	526		β per 1-SD	−0.057 (−0.155–0.042)	0.261
	GGT/ALP	24 h	517		β per 1-SD	−0.151 (−0.249– −0.053)	0.003*
	GGT/ALP	Delta	517		β per 1-SD	−0.137 (−0.233– −0.041)	0.005*
Non-overt DIC	ALBI	Admission	526	155	OR per 1-SD	2.43 (1.90–3.10)	<0.001*
	ALBI	24 h	517	150	OR per 1-SD	2.15 (1.69–2.75)	<0.001*
	ALBI	Delta	517	150	OR per 1-SD	0.83 (0.67–1.02)	0.078
	TBil/ALB	Admission	526	155	OR per 1-SD	1.75 (1.36–2.26)	<0.001*
	TBil/ALB	24 h	517	150	OR per 1-SD	2.17 (1.69–2.79)	<0.001*
	TBil/ALB	Delta	517	150	OR per 1-SD	1.24 (1.01–1.54)	0.043*
	DBil/TBil	Admission	526	155	OR per 1-SD	1.77 (1.40–2.24)	<0.001*
	DBil/TBil	24 h	517	150	OR per 1-SD	1.61 (1.30–2.00)	<0.001*
	DBil/TBil	Delta	517	150	OR per 1-SD	0.94 (0.77–1.16)	0.575
	GGT/ALP	Admission	526	155	OR per 1-SD	0.88 (0.70–1.11)	0.286
	GGT/ALP	24 h	517	150	OR per 1-SD	0.69 (0.52–0.91)	0.009*
	GGT/ALP	Delta	517	150	OR per 1-SD	0.77 (0.61–0.97)	0.026*
ATC	ALBI	Admission	526	166	OR per 1-SD	1.74 (1.40–2.16)	<0.001*
	ALBI	24 h	517	161	OR per 1-SD	1.72 (1.38–2.14)	<0.001*
	ALBI	Delta	517	161	OR per 1-SD	0.93 (0.76–1.14)	0.485
	TBil/ALB	Admission	526	166	OR per 1-SD	1.43 (1.14–1.79)	0.002*
	TBil/ALB	24 h	517	161	OR per 1-SD	1.63 (1.31–2.03)	<0.001*
	TBil/ALB	Delta	517	161	OR per 1-SD	1.14 (0.93–1.39)	0.206
	DBil/TBil	Admission	526	166	OR per 1-SD	1.71 (1.37–2.14)	<0.001*
	DBil/TBil	24 h	517	161	OR per 1-SD	1.65 (1.34–2.02)	<0.001*
	DBil/TBil	Delta	517	161	OR per 1-SD	1.00 (0.82–1.21)	0.968
	GGT/ALP	Admission	526	166	OR per 1-SD	0.83 (0.66–1.04)	0.104
	GGT/ALP	24 h	517	161	OR per 1-SD	0.70 (0.54–0.90)	0.006*
	GGT/ALP	Delta	517	161	OR per 1-SD	0.84 (0.68–1.03)	0.089

Model 3 adjusted for age, sex, admission lactate, admission pH, admission temperature, traumatic brain injury, hepatic injury, and surgical intervention records. *P < 0.05.

For the continuous ISTH DIC score measured 48 h after admission, baseline ALBI was strongly associated with a higher DIC score (*β* per 1-SD increase=0.470, 95% CI: 0.378–0.561; *P* < 0.001). The association remained evident for ALBI measured 24 h after admission (*β*=0.384, 95% CI: 0.292–0.477; *P* < 0.001), whereas the 24-hour change in ALBI showed a smaller inverse association (*β*=−0.116, 95% CI: −0.214 to −0.019; *P* = 0.020). TBil/ALB was also associated with the continuous DIC score at baseline, at 24 h after admission, and for the 24-hour change. DBil/TBil was associated with the DIC score at baseline and at 24 h, but not for its change value. By comparison, GGT/ALP showed no significant positive association at baseline; its 24-hour and change values were inversely associated with the DIC score.

For non-overt DIC assessed 48 h after admission, baseline ALBI was associated with substantially higher odds of the outcome (OR per 1-SD increase=2.43, 95% CI: 1.90–3.10; *P* < 0.001). A similar association was observed for ALBI measured 24 h after admission (OR = 2.15, 95% CI: 1.69–2.75; *P* < 0.001), while the 24-hour change in ALBI was not statistically significant. TBil/ALB and DBil/TBil were significantly associated with non-overt DIC at baseline and 24 h after admission; the 24-hour change in TBil/ALB showed a weaker but statistically significant association. GGT/ALP was not associated with non-overt DIC at baseline, although its 24-hour and change values showed inverse associations. For ATC assessed 48 h after admission, baseline ALBI (OR = 1.74, 95% CI: 1.40–2.16; *P* < 0.001) and 24-hour ALBI (OR = 1.72, 95% CI: 1.38–2.14; *P* < 0.001) were both significantly associated with increased odds of ATC, whereas the 24-hour change in ALBI was not. TBil/ALB and DBil/TBil at baseline and 24 h after admission were also associated with ATC, while GGT/ALP again showed an inverse association at 24 h. The inverse association observed for GGT/ALP should not be interpreted as protective, because a lower ratio may result from decreased GGT, increased ALP, or both; moreover, ALP is not liver-specific. The weaker association of *Δ*ALBI may partly reflect the inherent variability of a change score derived from two measurements and its mathematical dependence on the baseline value.

### 3.3 Comparison of predictive performance

Index-alone ROC analyses are displayed in Figure 1 and summarized in Table 3, with detailed estimates provided in Supplementary Table S3. For non-overt DIC assessed 48 h after admission, baseline ALBI showed the strongest discrimination among the baseline indices, with an AUC of 0.751 (95% CI: 0.704–0.794). The optimal cutoff was −1.964, yielding a sensitivity of 0.721 and a specificity of 0.697. ALBI measured 24 h after admission also showed moderate discrimination, with an AUC of 0.725 (95% CI: 0.680–0.766). Among the remaining indices, 24-hour TBil/ALB demonstrated relatively stronger performance for non-overt DIC (AUC=0.708, 95% CI: 0.663–0.753), whereas GGT/ALP showed weaker discrimination overall.

**Figure 1:**
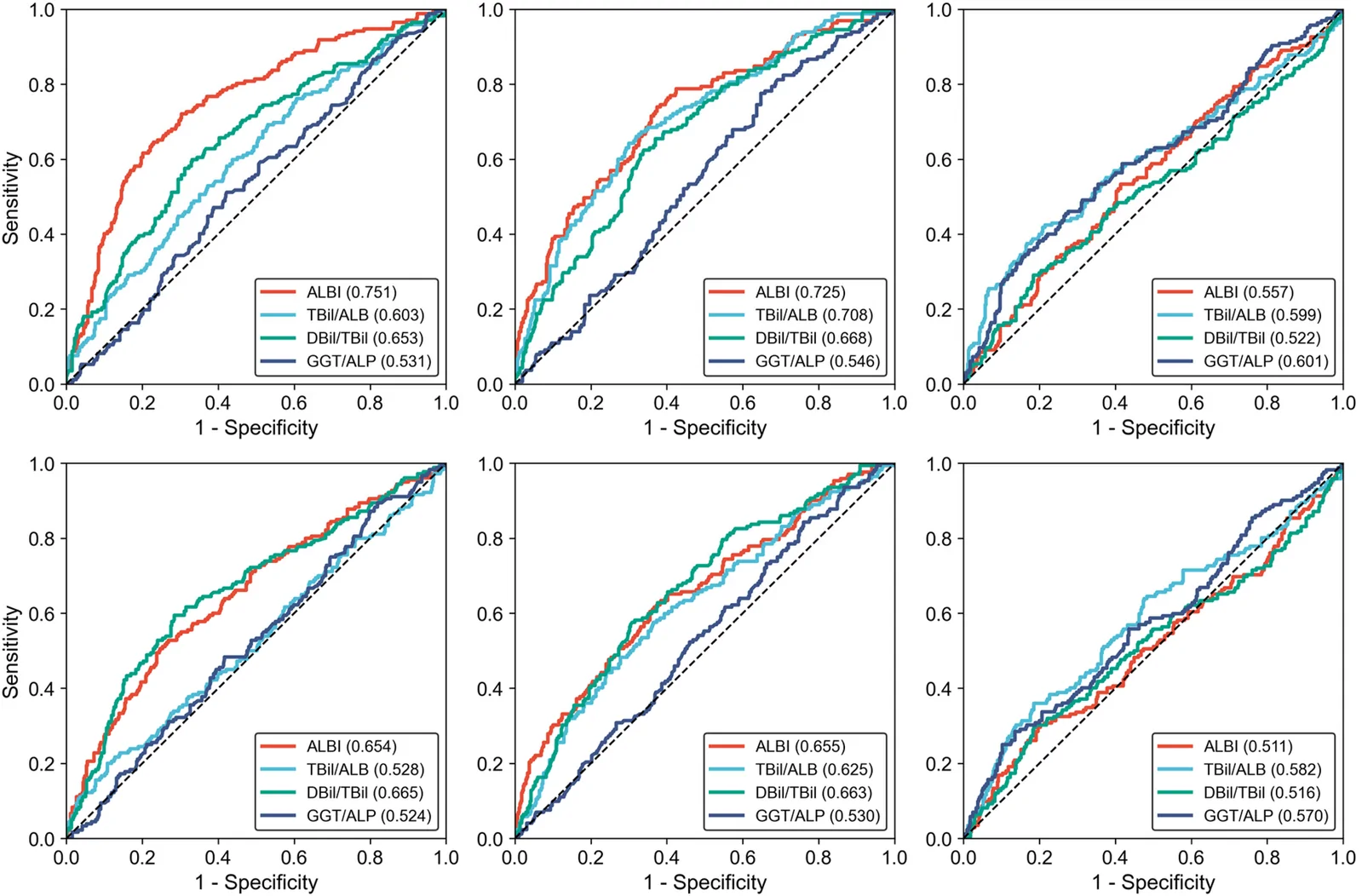
Discriminative performance of liver-function composite indices alone for TIC-related outcomes. Receiver operating characteristic curves showing the index-alone discriminative performance of ALBI, TBil/ALB, DBil/TBil, and GGT/ALP for non-overt DIC and ATC across admission, 24 h, and delta values*.*

**Table 3:** Discriminative performance of liver-function composite indices alone for non-overt DIC and ATC.

Outcome	Index	N/events	AUC (95% CI)	Youden cutoff	Sensitivity, %	Specificity, %
Non-overt DIC	ALBI	634/172	0.751 (0.704–0.794)	−1.964	72.1	69.7
	TBil/ALB	634/172	0.603 (0.553–0.653)	0.478	59.9	56.3
	DBil/TBil	634/172	0.653 (0.607–0.701)	0.385	59.9	66.9
	GGT/ALP	634/172	0.531 (0.487–0.581)	0.390	51.2	57.8
ATC	ALBI	634/180	0.654 (0.603–0.700)	−1.887	52.8	73.1
	TBil/ALB	634/180	0.528 (0.480–0.573)	0.766	20.0	89.2
	DBil/TBil	634/180	0.665 (0.619–0.710)	0.391	59.4	71.6
	GGT/ALP	634/180	0.524 (0.477*–*0.571)	0.205	89.4	18.1

ROC analyses used each admission liver-function composite index as a standalone marker, without covariate adjustment. Cutoffs were selected using the Youden index and should be interpreted as exploratory thresholds rather than clinical decision cutoffs. Detailed ROC analyses across 24 h and delta measures are provided in Supplementary Table S3. ALBI, albumin–bilirubin; ATC, acute traumatic coagulopathy; AUC, area under the receiver operating characteristic curve; CI, confidence interval; DIC, disseminated intravascular coagulation; ROC, receiver operating characteristic; TIC, trauma-induced coagulopathy.

For ATC assessed 48 h after admission, AUCs were generally lower than those observed for non-overt DIC. Baseline ALBI yielded an AUC of 0.654 (95% CI: 0.603–0.700), and ALBI measured 24 h after admission yielded an AUC of 0.655 (95% CI: 0.598–0.708). DBil/TBil showed comparable discrimination for ATC at baseline and 24 h after admission. Incremental ROC analyses are reported in Supplementary Table S4. For non-overt DIC, the clinical model alone achieved an AUC of 0.686; this increased to 0.776 after the addition of baseline ALBI. Similar improvements were observed for ATC and overt DIC when baseline ALBI was added to the clinical model. The stronger observed performance of baseline ALBI relative to ΔALBI suggests that the admission value may better capture underlying physiological vulnerability. By contrast, short-term changes are more readily influenced by early treatment and measurement variability.

### 3.4 Subgroup analysis

Exploratory interaction analyses evaluating the association between baseline ALBI and non-overt DIC are presented in Figure 2 and Supplementary Table S5. The examined subgroup variables included sex, age group, admission lactate level, traumatic brain injury, hepatic injury, surgery, admission hypothermia, and admission acidosis. Most interaction terms were not statistically significant. A nominal interaction was observed for acidosis (*P* for interaction = 0.024), but it did not remain significant after either Bonferroni or Benjamini–Hochberg false-discovery-rate correction (both adjusted *P* = 0.194). No other significant interactions were identified. The association between baseline ALBI and non-overt DIC remained positive across the examined subgroups, supporting the consistency of the association across different clinical strata.

**Figure 2:**
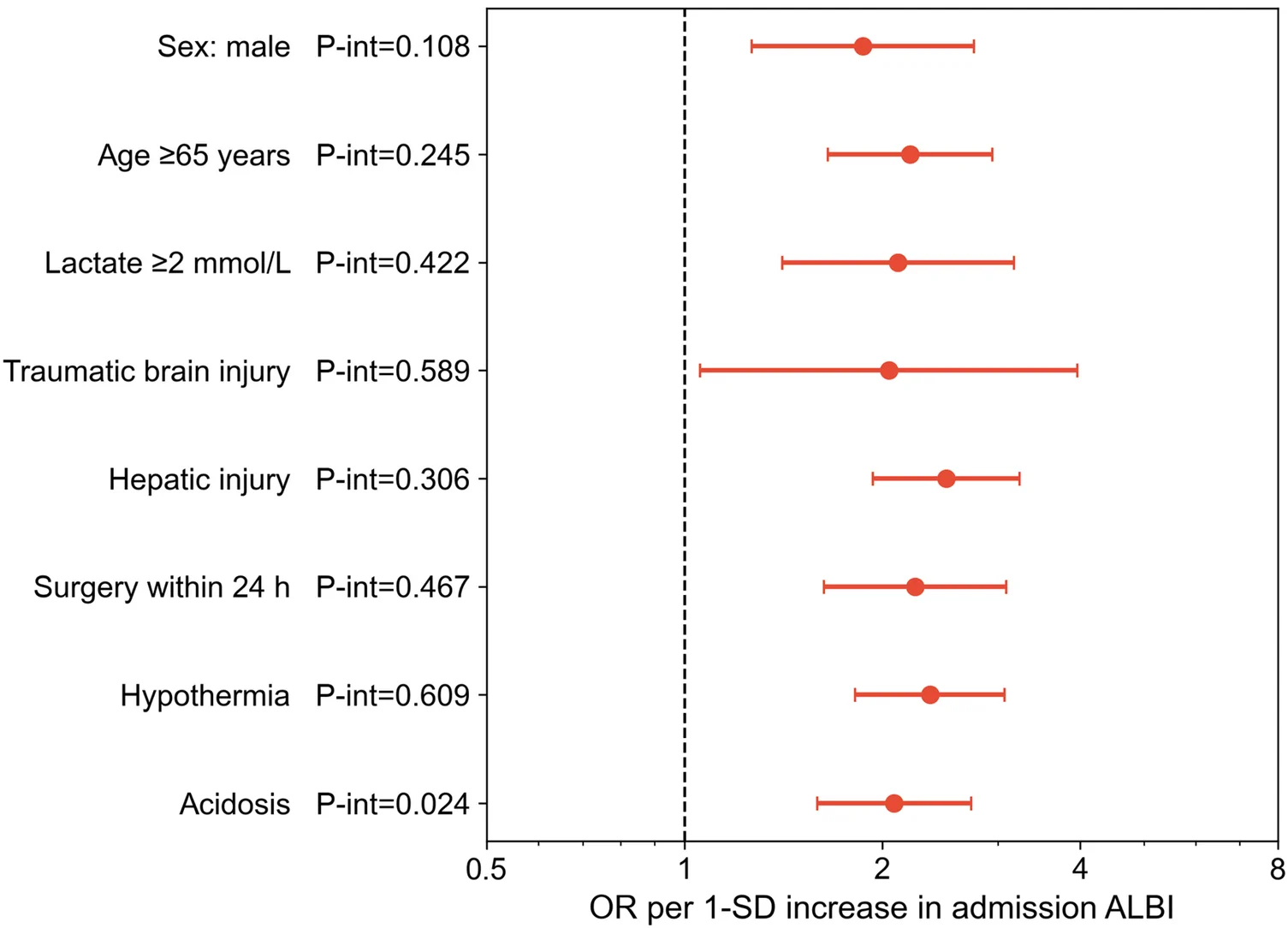
Exploratory interaction analysis for admission ALBI and non-overt DIC. Forest plot showing exploratory interaction analyses for the association between admission ALBI and non-overt DIC. Odds ratios are expressed per 1-SD increase in admission ALBI.

### 3.5 Sensitivity analyses

Sensitivity analyses are summarized in Figure 3 and Supplementary Table S6. In the main complete-case analysis, baseline ALBI was associated with non-overt DIC assessed 48 h after admission (OR = 2.43, 95% CI: 1.90–3.10; *P* < 0.001), ATC assessed 48 h after admission (OR = 1.74, 95% CI: 1.40–2.16; *P* < 0.001), and the continuous ISTH DIC score measured 48 h after admission (*β*=0.470, 95% CI: 0.378–0.561; *P* < 0.001). These associations were maintained after excluding patients with severe baseline coagulopathy. In that restricted analysis, baseline ALBI remained associated with non-overt DIC (OR = 2.30, 95% CI: 1.79–2.95; *P* < 0.001), ATC (OR = 1.69, 95% CI: 1.36–2.11; *P* < 0.001), and the ISTH DIC score (*β*=0.426, 95% CI: 0.335–0.517; *P* < 0.001). Results were also consistent in the single-imputation analysis, where baseline ALBI remained associated with non-overt DIC (OR = 2.58, 95% CI: 2.04–3.26; *P* < 0.001), ATC (OR = 1.79, 95% CI: 1.46–2.20; *P* < 0.001), and the ISTH DIC score (*β*=0.527, 95% CI: 0.438–0.616; *P* < 0.001). Associations for ALBI measured 24 h after admission were similarly preserved across sensitivity analyses, whereas the 24-hour change in ALBI showed weaker and less consistent associations. Overall, ALBI showed consistent positive associations at admission and 24 h and had the highest admission index-alone AUC for non-overt DIC. TBil/ALB also showed positive associations, with stronger index-alone discrimination at 24 h than at admission. DBil/TBil was associated with outcomes at the fixed time points and showed discrimination comparable to ALBI for ATC. GGT/ALP showed weaker index-alone discrimination and inverse adjusted associations at 24 h. Changes over the first 24 h were less consistently associated with the binary outcomes. Collectively, these descriptive comparisons suggest index- and outcome-specific patterns of discrimination, rather than a consistent ranking of predictive performance across all endpoints.

**Figure 3:**
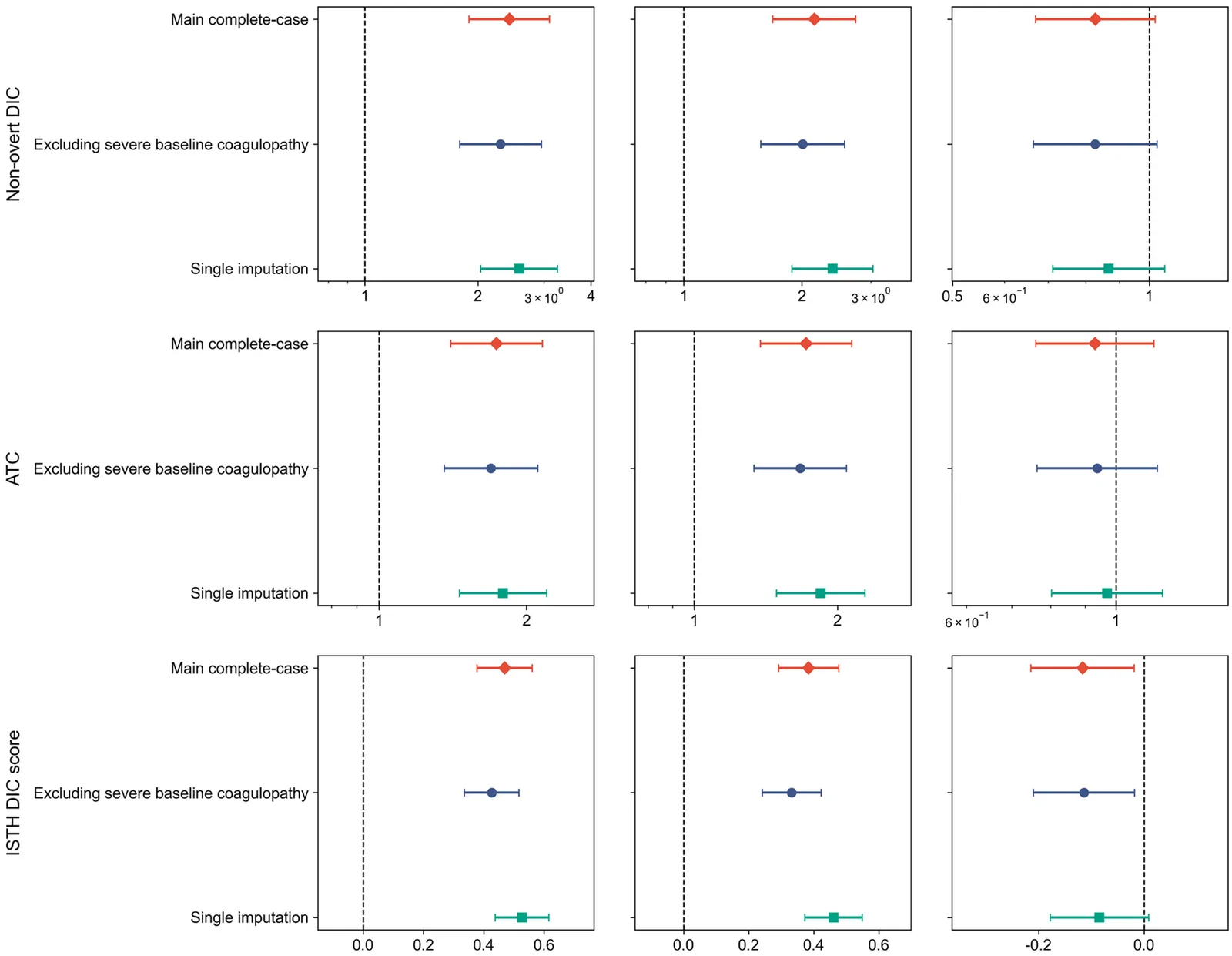
Sensitivity analyses for ALBI across TIC-related outcomes. Forest plot summarizing the sensitivity analyses of ALBI across TIC-related outcomes, including non-overt DIC, ATC, and the continuous ISTH DIC score. Analyses were repeated across admission, 24 h, and delta ALBI values under the main complete-case model, exclusion of severe baseline coagulopathy, and single-imputation analysis.

## 4 Discussion

In this adult trauma cohort, composite liver-function indices, particularly ALBI, were associated with TIC-related outcomes assessed 48 h after admission. Several findings deserve emphasis. First, the ALBI score showed the most robust and consistent associations with all TIC-related outcomes, including the continuous ISTH DIC score, non-overt DIC defined operationally in this study as an ISTH score ≥ 4, and ATC defined by PTr > 1.2. Second, the TBil/ALB ratio also demonstrated independent predictive value, but its overall performance was slightly less stable than that of ALBI. Third, DBil/TBil and GGT/ALP showed less consistent associations across outcomes and time points. Finally, dynamic changes over the first 24 h provided additional information, but their role was supplementary rather than equivalent to baseline assessment. Taken together, these findings suggest that ALBI may be the most stable adjunctive liver-function-related risk marker among the evaluated indices, but it should not be interpreted as a standalone diagnostic or treatment-guiding tool.

The observed association between ALBI and TIC-related outcomes is biologically plausible but should be interpreted cautiously in the acute trauma setting. TIC is a multifactorial syndrome driven by tissue injury, shock, hypoperfusion, systemic inflammation, endothelial dysfunction, and dysregulated fibrinolysis (1). Because the liver contributes to the synthesis of procoagulant factors, endogenous anticoagulants, and fibrinolytic regulators, liver-function-related abnormalities may be associated with impaired hemostatic adaptation during acute traumatic stress (12). ALBI may capture a composite liver-function-related and systemic stress phenotype because it combines albumin, which is influenced by synthetic function, inflammation, nutritional status, hemodilution, and capillary leak, with bilirubin, which may reflect excretory dysfunction, hypoperfusion, hemolysis, and transfusion-related changes (8, 13, 14). In the acute trauma setting, lower albumin may also indicate reduced physiologic reserve and heightened capillary leak, whereas elevated bilirubin may reflect hypoperfusion-related hepatocellular dysfunction and systemic oxidative stress (13, 14). By combining these two components in a weighted formula, ALBI may provide a more stable composite marker than simpler ratios derived from the same analytes (8).

An additional strength of ALBI is its temporal stability. In our cohort, ALBI remained relatively stable at the population level during the early post-trauma period, whereas conventional coagulation parameters changed substantially over time. This pattern suggests that ALBI is not simply mirroring short-term fluctuations in coagulation parameters, although the present observational data cannot determine whether ALBI reflects an upstream predisposition or an accompanying systemic response. A worse baseline ALBI score may help identify patients at higher risk of subsequent TIC-related abnormalities assessed 48 h after admission, but it should not be interpreted as proof of limited hepatic reserve. Once these patients are exposed to ongoing hemorrhage, hemodilution, acidosis, hypothermia, and inflammatory activation, reduced capacity to sustain coagulation factor homeostasis may contribute to subsequent TIC progression (1, 15). This interpretation is clinically important because it positions ALBI as a baseline vulnerability marker rather than a dynamic substitute for conventional coagulation monitoring.

The performance of the TBil/ALB ratio further supports the importance of bilirubin-albumin–based composite assessment. TBil/ALB consistently showed independent associations with TIC-related outcomes and emerged as the second-best marker in our analyses. From a practical perspective, this ratio has the advantage of being extremely simple to calculate, which may facilitate bedside use in settings where rapid computational tools are not readily available. Previous studies have also shown that bilirubin-to-albumin–based indices carry prognostic value in critically ill patients without established hepatobiliary disease. More recently, Shah et al. demonstrated that an elevated bilirubin-to-albumin ratio was independently associated with increased mortality in patients with liver diseases, further supporting the prognostic utility of this composite index across diverse clinical populations (16). However, compared with ALBI, TBil/ALB appeared less stable across models and outcomes. A likely explanation is that the simple ratio does not account for the differential contribution of albumin and bilirubin as effectively as the weighted ALBI formula (8). Thus, while TBil/ALB may still be clinically useful as a pragmatic alternative, especially in resource-limited settings, our results indicate that it should be viewed as a secondary option rather than the preferred marker.

In contrast, DBil/TBil and GGT/ALP showed limited and inconsistent predictive utility. For DBil/TBil, some models yielded large odds ratios, but these were typically accompanied by very wide confidence intervals, indicating substantial statistical uncertainty. Such patterns suggest instability rather than robust predictive signal, and they warrant cautious interpretation. Mechanistically, the proportion of direct bilirubin may be influenced by processes that are less specific to hepatic reserve in acute trauma, including hemolysis, hematoma resorption, and transient metabolic disturbances (17). Similarly, the GGT/ALP ratio is more closely related to hepatobiliary enzyme patterns than to the synthetic and regulatory liver functions most relevant to coagulation homeostasis (18). In trauma patients, these enzymes may also be affected by alcohol exposure, medications, systemic inflammation, and related factors, further reducing specificity (18). Our results therefore suggest that not all composite liver-related biomarkers are equally informative for TIC, and indices based on enzymatic injury or bilirubin fractionation may provide less stable information than ALBI for TIC-related risk assessment.

The findings regarding 24-hour changes also deserve careful interpretation. Dynamic changes in ALBI and TBil/ALB were associated with later TIC-related burden, indicating that early hepatic trajectory contains clinically relevant information. Nevertheless, the magnitude and consistency of these associations did not surpass those of baseline ALBI. This distinction is important for clinical application. Delta values should not be considered competing primary predictors, but rather supplementary monitoring indicators that may refine ongoing risk assessment after admission. These findings support a descriptive framework in which baseline ALBI and 24-hour changes provide complementary information, with baseline ALBI showing the more consistent association pattern. This interpretation is consistent with the broader principle that baseline physiologic reserve and short-term trajectory provide different, but complementary, prognostic information (19).

Our results also have direct translational relevance. ALBI and TBil/ALB are derived from total bilirubin and albumin, two routine laboratory parameters that are widely available, inexpensive, and rapidly obtained in standard clinical practice. Because albumin and bilirubin are already measured in most trauma patients, calculating ALBI requires no additional blood draw, specialized assay, or equipment cost. Unlike specialized coagulation assays, these indices could be readily evaluated in future trauma risk-stratification studies, particularly in settings where advanced viscoelastic testing is unavailable or delayed (20, 21). In the same complete-case sample, adding admission ALBI to the clinical model increased the AUC for non-overt DIC from 0.686 to 0.776, suggesting that ALBI captures prognostic information beyond routine clinical variables. Cross-study comparison with existing TIC prediction approaches is limited by differences in populations, outcome definitions, and assessment windows; therefore, our results should be interpreted as evidence that ALBI can refine risk ranking within this cohort rather than as proof of superiority over established tools. Importantly, the predictive value of ALBI was observed at admission, before later TIC-related outcomes became apparent. Its potential clinical role is therefore to help identify patients who may warrant earlier or more intensive coagulation monitoring, rather than to guide transfusion or resuscitation decisions. Whether ALBI-guided monitoring or management improves outcomes requires prospective evaluation (22).

Our study adds to the growing literature linking liver-function-related markers to outcomes after trauma, but it extends that concept into the specific context of TIC. Previous studies have shown that ALBI-based indices are associated with prognosis in trauma and other populations (23), yet direct comparisons among multiple composite liver function indices for TIC-related outcomes have been lacking. By focusing on comparative performance and temporal behavior, our study helps clarify which liver-related composite marker is most suitable for early trauma use. The central message is not simply that liver dysfunction matters, but that the way it is summarized matters: an index reflecting integrated hepatic reserve performs better than markers that are either overly simplified or biologically less specific to coagulation-related liver function.

Several limitations should be acknowledged. First, the retrospective single-center design limits causal inference and external generalizability and may have introduced residual confounding. Second, although the analytic cohort was expanded, the number of overt DIC events remained limited, and results for overt DIC should therefore be interpreted cautiously. Non-overt DIC, defined as ISTH score ≥4, was analyzed as an exploratory early coagulopathy phenotype, whereas overt DIC defined as ISTH score ≥5 remains the standard ISTH binary DIC outcome (24). Third, patients with severe baseline coagulopathy were retained in the primary analysis to reduce spectrum restriction, and exclusion of these patients was performed only as a sensitivity analysis. Fourth, we did not directly compare these indices with viscoelastic assays such as TEG or ROTEM, which remain important tools in contemporary TIC assessment (20, 21). Fifth, ALBI values measured 24 h after admission and 0–24-hour changes may reflect evolving clinical status after resuscitation, surgery, or other interventions rather than baseline risk alone. Sixth, platelet counts at T1 and T2 were not measured in 492 of 634 patients (77.6%); however, T3 platelet counts were complete, and the primary analyses were based on liver-function composite indices and T3 outcomes, so this missingness did not affect the main conclusions. Future prospective multicenter studies should validate these findings, assess incremental value beyond established coagulation testing, and determine whether ALBI-guided early risk stratification can improve transfusion strategy or clinical outcomes.

In conclusion, among the four composite liver-function indices evaluated, ALBI showed the most consistent association pattern and the strongest index-alone discrimination for non-overt DIC in adult trauma patients. TBil/ALB may serve as a useful secondary alternative, whereas DBil/TBil and GGT/ALP showed less stable associations and weaker discrimination across TIC-related outcomes. Dynamic changes over the first 24 h may provide additional monitoring value, but they should be interpreted as complementary to, rather than replacements for, baseline assessment. These findings suggest that ALBI may serve as a simple adjunctive risk marker for TIC-related outcomes, but prospective multicenter validation with more complete resuscitation and treatment data is needed before clinical implementation.

## 5 Conclusion

In adult trauma patients, baseline ALBI and ALBI measured 24 h after admission were consistently associated with TIC-related outcomes assessed 48 h after admission, including the continuous ISTH DIC score, non-overt DIC, and ATC. Among the evaluated liver-function composite indices, ALBI showed the most stable overall performance and the strongest discrimination for non-overt DIC. Subgroup analyses revealed no statistically significant interaction after multiplicity correction, indicating that the association between baseline ALBI and non-overt DIC was consistent across the examined clinical subgroups. These findings suggest that ALBI may serve as a readily available adjunctive risk marker for early coagulopathy risk stratification, although prospective validation remains warranted.

## Data Availability

The raw data supporting the conclusions of this article will be made available by the authors, without undue reservation.
